# Experiments in Artificial Theory of Mind: From Safety to Story-Telling

**DOI:** 10.3389/frobt.2018.00075

**Published:** 2018-06-26

**Authors:** Alan F. T. Winfield

**Affiliations:** Bristol Robotics Laboratory, University of the West of England, Bristol, United Kingdom

**Keywords:** anticipation, simulation-based internal models, theory-of-mind, cognitive robotics, multi-robot systems, human-robot interaction, social intelligence, machine consciousness

## Abstract

Theory of mind is the term given by philosophers and psychologists for the ability to form a predictive model of self and others. In this paper we focus on synthetic models of theory of mind. We contend firstly that such models—especially when tested experimentally—can provide useful insights into cognition, and secondly that artificial theory of mind can provide intelligent robots with powerful new capabilities, in particular social intelligence for human-robot interaction. This paper advances the hypothesis that simulation-based internal models offer a powerful and realisable, theory-driven basis for artificial theory of mind. Proposed as a computational model of the simulation theory of mind, our simulation-based internal model equips a robot with an internal model of itself and its environment, including other dynamic actors, which can test (i.e., simulate) the robot's next possible actions and hence anticipate the likely consequences of those actions both for itself and others. Although it falls far short of a full artificial theory of mind, our model does allow us to test several interesting scenarios: in some of these a robot equipped with the internal model interacts with other robots without an internal model, but acting as proxy humans; in others two robots each with a simulation-based internal model interact with each other. We outline a series of experiments which each demonstrate some aspect of artificial theory of mind.

## 1. Introduction

Theory of mind is the term given by philosophers and psychologists for the ability to predict the actions of self and others (Carruthers and Smith, 1996). With theory of mind, it is supposed, we are able to anticipate how others might behave in particular circumstances. However, the idea of theory of mind is empirically weak—we have only a poor understanding of the neurological or cognitive processes that give rise to theory of mind. Artificial Intelligence (AI), and its embodied counterpart—robotics, provides a powerful synthetic approach to theory of mind because it allows us to ask the question “how would we build artificial theory of mind in a robot?” and opens the possibility that we could test theories of theory of mind.

The role of theory of mind in consciousness (or, indeed of consciousness in theory of mind) is both unclear and controversial (Carruthers, 2009; Sebastian, 2016). In this paper we avoid this difficult question by focusing instead on synthetic models of theory of mind. We contend firstly that such models—especially when tested experimentally—can provide valuable insights into both natural and artificial cognition, and secondly that artificial theory of mind can provide intelligent robots with powerful new capabilities, in particular social intelligence for human-robot interaction. Artificial theory of mind has been recently highlighted as one of the *Grand Challenges of Science Robotics*: “The three most significant challenges that stem from building robots that interact socially with people are modeling social dynamics, learning social and moral norms, and building a robotic theory of mind” (Yang et al., 2018).

The aim of this paper is to advance the hypothesis that simulation-based internal models offer a powerful and realizable, theory-driven basis for artificial theory of mind. Proposed as a computational model of the simulation theory of mind (Goldman, 2006), our simulation-based internal model equips a robot with an internal model of itself and its environment, including other dynamic actors, which can test (i.e., simulate) the robot's next possible actions and hence anticipate the likely consequences of those actions both for itself and others; importantly our simulation-based internal model is a practical proposition with current technology. Although it falls far short of a full artificial theory of mind, our model allows us to test several interesting scenarios: in some of these a robot equipped with the internal model interacts with other robots, without an internal model but acting as proxy humans; in others two robots each with a simulation-based internal model interact with each other. We are able to predict second and third order interactions^1^ and, in some cases, observe interesting and unexpected emergent behaviors.

This paper proceeds as follows. First in section 2 we adopt a working definition of theory of mind and outline theories of theory of mind. Choosing the simulation theory of mind (ST) we then outline the conceptual basis for simulation-based internal models, together with prior work which uses such models, before proposing a generic computational model of ST. Section 3 then introduces a series of experiments in (simple) artificial theory of mind: in the first the aim is improved *safety*; in the second it is simple *ethical* behaviors—including a scenario in which the ethical robot faces a dilemma; in the third one robot aims to infer the goals of another to rationally *imitate* it. The fourth and final exemplar is a thought experiment which outlines a proposal for an embodied computational model of *storytelling*, using robots. It is important to note that none of these experiments were conceived as a solution to the problem of artificial theory of mind. It was only *post-hoc* that we recognized that—since each experiment involves one or more robots which predict the behavior of others—taken together they offer some insight into practical artificial theory of mind. Take ethical robots as a case in point. Although a robot may not need theory of mind to behave ethically it is easy to see that the ability to predict the intentions of others would greatly facilitate and likely extend the scope of its ethical responses^2^. Section 4 concludes the paper with a discussion which both draws high-level conclusions from the experimental work outlined in section 3 and makes the case that this work does demonstrate a number of components of theory of mind and can therefore reasonably be described as “experiments in artificial theory of mind.”

## 2. From simulation theory to a simulation-based internal model

### 2.1. Theories of theory of mind

One difficulty of this paper is that there is no single definition of theory of mind and its attributes. Definitions vary according to the context so, in animal cognition, for instance, Roberts (2001) writes “The term theory of mind refers to the fact that people know about minds …the inferences you make about others minds may often guide your behavior,” whereas Breed and Moore (2012) write “An animal with a theory of mind can form hypotheses about the thoughts of surrounding animals.” In child development theory of mind refers to “childrens understanding of people as mental beings, who have beliefs, desires, emotions, and intentions” (Astington and Dack, 2008), with mental representation and false belief regarded as key components. And in Birch et al. (2017) “Perspective taking, or theory of mind, involves reasoning about the mental states of others (e.g., their intentions, desires, knowledge, beliefs) and is called upon in virtually every aspect of human interaction.” In this paper we resolve this difficulty by settling on “to explain and predict the actions, both of oneself, and of other intelligent agents” as our working definition of theory of mind (Carruthers and Smith, 1996).

There are a number of theories of theory of mind (Carruthers and Smith, 1996); and such theories are generally grouped into two broad categories, known as theory theory (TT) and simulation theory (ST)^3^. For a good outline comparison of TT and ST see Michlmayr (2002). Theory theories hold that one intelligent agent's understanding of another's mind is based on innate or learned rules, sometimes known as folk psychology. In TT these hidden rules constitute a “theory” because they can be used to both explain and make predictions about others' intentions. In contrast “simulation theory suggests that we do not understand others through the use of a folk psychological theory. Rather, we use our own mental apparatus to form predictions and explanations of someone by putting ourselves in the shoes of another person and simulating them” (Michlmayr, 2002). Goldman (2006) introduces the idea of mental simulation: “the simulation of one mental process by another mental process,” and makes the important distinction between intra personal and interpersonal mental simulation; the former is simulation of self, and the latter the simulation of other. Goldman (2006) also marks the distinction between computational modeling simulation and replication simulation, noting that only the latter is of interest to theory of mind; we would contend that the former is of great interest to *artificial* theory of mind.

In this paper we adopt ST as both the inspiration and theoretical basis for our hypothesis that simulation-based internal models offer a powerful approach to building artificial theory of mind, not because we have a principled theoretical preference for ST over TT, but because simulation-based internal models provide a realizable computational model for ST.

Also relevant here is the simulation theory of cognition (Hesslow, 2002; Wilson, 2002). This theory hypothesizes that cognitive introspection utilizes the same processes as interaction with the external environment. During introspection (thinking), actions are covert and are assumed to generate, via associative brain mechanisms, the sensory inputs that elicit further actions (Hesslow, 2012). In this view, cognition requires a grounded representation of the world that is not composed of abstract symbols; a simulation provides just such a model.

### 2.2. Simulation-based internal modeling

A simulation-based internal model is a mechanism for internally representing both the system and its current environment. If we embed a simulation of a robot, including its currently perceived environment, inside that robot then the robot has a “mechanism for generating and testing *what-if* hypotheses; i.e.,

1. *what if* I carry out action *x*.? and, …
2. of several possible next actions *x*_*i*_, *which* should I choose?" (Winfield, 2014)

Holland writes: an Internal Model allows a system to look ahead to the future consequences of current actions, without actually committing itself to those actions (Holland, 1992, p. 25). This leads to the idea of “an internal model as a *consequence engine*—a mechanism for predicting and hence anticipating the consequences of actions” (Winfield and Hafner, 2018).

The idea of embedding a simulator of a robot within that robot is not new, but implementation is technically challenging, and there have been relatively few examples described in the literature. One notable example is within the emerging field of machine consciousness (Holland, 2003; Holland and Goodman, 2003). Marques and Holland (2009) define a “functional imagination” as “a mechanism that allows an embodied agent to simulate its own actions and their sensory consequences internally, and to extract behavioral benefits from doing so”; a embedded simulation-based internal model provides such a mechanism.

Bongard et al. (2006) describe a 4-legged starfish like robot that makes use of explicit internal simulation, both to enable the robot to learn it's own body morphology and control, and notably allow the robot to recover from physical damage by learning the new morphology following the damage. The internal model of Bongard et al. models only the robot, not its environment. See also Zagal and Lipson (2009). In contrast Vaughan and Zuluaga (2006) demonstrate self-simulation of both a robot and its environment in order to allow a robot to plan navigation tasks with incomplete self-knowledge; their approach significantly provides perhaps the first experimental proof-of-concept of a robot using self-modeling to anticipate and hence avoid unsafe actions. Zagal et al. (2009) describe self-modeling using internal simulation in humanoid soccer robots; in what they call a ‘back-to-reality' algorithm, behaviors adapted and tested in simulation are transferred to the real robot.

In robotics advanced physics and sensor-based simulation tools are routinely used to model, develop or evolve robot control algorithms prior to real-robot tests. Well-known robot simulators include Webots (Michel, 2004), Gazebo (Koenig and Howard, 2004), Player-Stage (Vaughan and Gerkey, 2007), and V-REP (Rohmer et al., 2013). Simulation technology is now sufficiently mature to provide a practical route to implementation of an embedded simulation-based internal model. Furthermore Stepney (2018) sets out a principled approach to simulation which treats a simulator as a scientific instrument.

### 2.3. A computational model of simulation theory of mind

**Figure 1:**
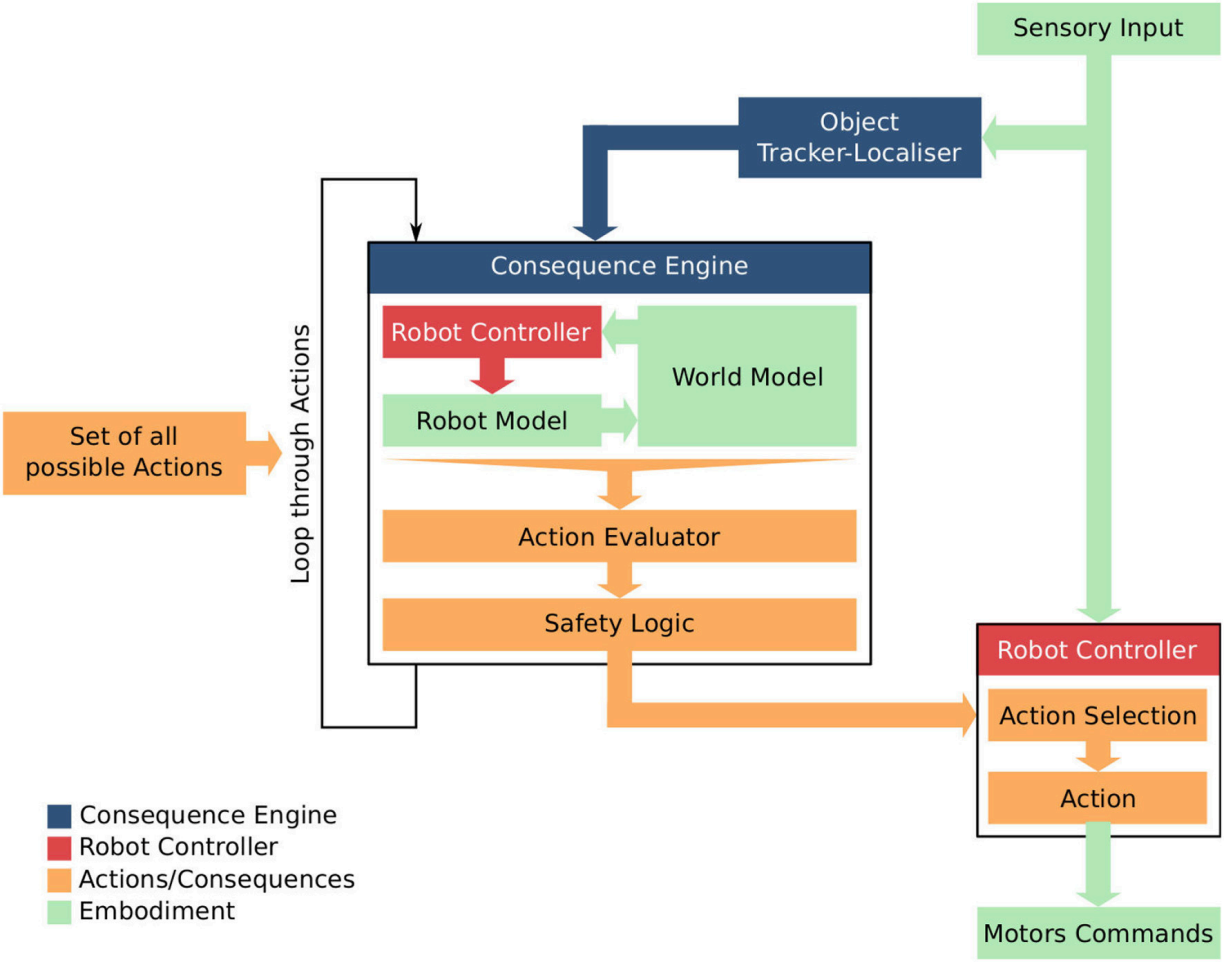
The Consequence Engine: an architecture for robot anticipation using a simulation-based internal model. Figure from Blum et al. (2018).

We have recently proposed an architecture for a robot with a simulation-based internal model which is used to test and evaluate the consequences of that robot's next possible actions. Shown in Figure 1 “the machinery for modeling next actions is relatively independent of the robot's controller; the robot is capable of working normally without that machinery, albeit without the ability to generate and test what-if hypotheses. The what-if processes are not in the robot's main control loop, but instead run in parallel to moderate the Robot Controller's normal action selection process, acting in effect as a kind of governor” (Blum et al., 2018). This governance might be to rule out certain actions because they are modeled as unsafe for the robot, or to recommend new robot actions to, for instance, prevent an accident.

“At the heart of the architecture is the Consequence Engine. The CE is initialized from the Object Tracker-Localizer, and loops through all possible next actions; these next actions are generated within the Robot Controller (RC) and transferred to the mirror RC within the CE (for clarity this data flow is omitted from Figure 1). For each candidate action the CE simulates the robot executing that action, and generates a set of model outputs ready for evaluation by the Action Evaluator. The Consequence Evaluator loops through each possible next action; this is the Generate-and-Test loop. Only when the complete set of next possible actions has been tested does the Consequence Evaluator send, to the Robot Controller, its recommendations” (Winfield et al., 2014). These processes are explained in detail in Blum et al. (2018).

We argue that the architecture outlined here represents a computation model of artificial theory of mind. First, the model clearly provides a robot with the ability to self-model and hence predict the consequences of its own actions. Second the model can be used to predict another dynamic agent's actions and—if they interact—the consequences of this robot's actions to that other agent. This predictive modeling of others can be implemented in two ways depending on the way we model those other agents.

1. In the first, which we can call the ST-self plus TT-other (ST+TT) model, the other dynamic agents (i.e., robots) are modeled within the World Model of this robot using simple theory, for example a ballistic model for moving agents. Since this variant combines elements of ST and TT it models a hybrid theory of mind.
2. In the second, which we can call ST-self plus ST-other (ST+ST), the whole of the consequence engine can be initialized for the other agent and run introspectively, recalling the simulation theory of cognition (Hesslow, 2012). Here the robot models each other agent *exactly* as it models itself, i.e., as a conspecific. This variant models pure ST^4^.

The experiments outlined in the next section illustrate both ST+TT and ST+ST variants.

## 3. Experiments in artificial theory of mind

### 3.1. Safety: the corridor experiment

We have implemented and tested the simulation-based internal model architecture outlined above in an experimental scenario, which we call the corridor experiment (Blum et al., 2018). Inspired by the problem of how mobile robots could move quickly and safely through crowds of moving humans, the aim of this experiment is to compare the performance of our simulation-based internal model with a purely reactive approach. In other words: can a robot's safety be improved with simple artificial theory of mind?

**Figure 2:**
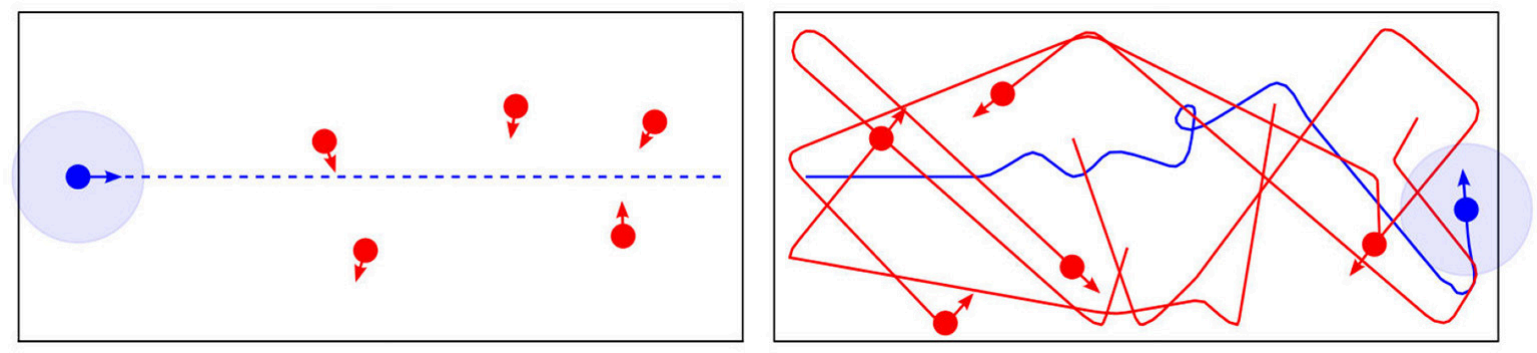
The corridor experiment goal **(left)**, with 5 (red) robots moving randomly and one intelligent (CE) robot (blue) with a simulation-based internal model. **(Right)** shows (simulated) trajectories of all six robots by the time blue has reaching the end of the corridor. Figure from Blum et al. (2018).

**Figure 3:**
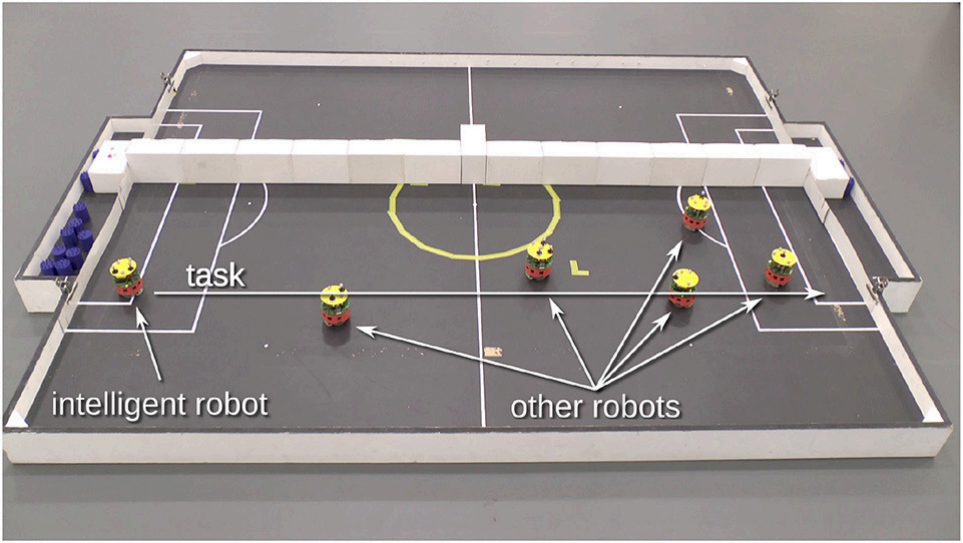
The corridor experiment, using e-puck robots (Mondada et al., 2009) fitted with Linux extension boards (Liu and Winfield, 2011). This image shows the initial condition with the CE (intelligent) robot on the left and the five proxy-human robots positioned at randomly selected locations in the corridor. The arena markings have no significance here. Figure from Blum et al. (2018).

In this experiment one mobile robot (the CE-robot) is equipped with the consequence engine of Figure 1, while 5 other mobile robots have only simple obstacle avoidance behaviors. The setup is shown in Figure 2 (left); here the smart CE-robot is shown in blue at its starting position. The CE-robot's goal is to reach the end of the corridor on the right while maintaining its own safety by avoiding—while also maintaining a safe distance—the five proxy-human robots shown in red. Figure 2 (right) shows the trajectories of all six robots during a simulated run of the experiment, with the CE-robot reaching the end of the corridor. Figure 3 shows the real-robot experimental setup.

In this experiment the CE robot models each of the proxy-human robots as a ballistic agent with obstacle avoidance—in other words as agents that will continue to move in their current direction and speed unless confronted with an obstacle, which may be another agent or the corridor wall. The CE runs in real-time and is updated every 0.5 s with the actual position and direction of the proxy-humans within the CE robot's attention radius. This is not an unreasonable model when considering how you might behave when avoiding another person who is not paying attention to where they are going—peering at their smartphone perhaps.

Results of the corridor experiment (detailed in Blum et al., 2018) show that for a relatively small cost in additional distance covered, the likelihood that a proxy-human robot comes within the CE-robot's safety radius falls to zero. Clearly there is a computational cost. This is entirely to be expected: anticipatory modeling of other agents clearly incurs a computational overhead.

In the corridor experiment there is an asymmetry: the CE-robot has a model for the proxy-human robots whereas they have no model for the CE-robot. In an extension to the corridor experiment which we call the pedestrian experiment two robots—each equipped with the same CE—approach each other. As with the corridor experiment each models the other as a simple ballistic agent but here we have symmetry with each agent paying full attention to the other, trying to anticipate how it might behave and planning its own actions accordingly. Is it possible that our “pedestrian” robots might, from time to time, engage in the kind of “dance” that human pedestrians do when one steps to their left and the other to their right only to compound the problem of avoiding a collision with a stranger?

**Figure 4:**
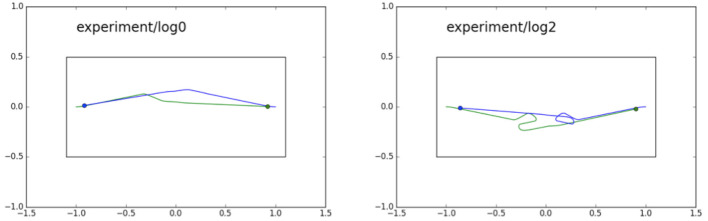
The pedestrian experiment—two trials showing robot trajectories. Two robots, blue and green, are each equipped with a CE. Blue starts from the right, with a goal position on the left, while at the same time green starts from the left with a goal position on the right. **(Left)** We see the typical behavior in which the two robots pass each other without difficulty, normally because one robot—anticipating a collision—changes direction first, in this case green. **(Right)** Here both robots make a decision to turn at the same time, green to its left and blue to its right; a “dance” then ensues before the impasse is resolved.

Results show that we do indeed observe this interesting emergent behavior. In five experimental runs four resulted in the two pedestrian robots passing each other by both turning either to the left or to the right—Figure 4 (left) shows one example of this behavior. However, in one run, shown in Figure 4 (right) we observe a brief dance caused when both robots decide, at the same time, to turn toward each other—each predicting wrongly that the other robot would continue its currently trajectory—before the two robots resolve the impasse and pass each other safely.

### 3.2. Toward ethical robots

We have conducted exploratory work—based on the same simulation-based internal model architecture outlined in section 2—to explore the possibility of robots capable of making decisions based on ethical rules. These robots implement simple consequentialist ethics with rules based on Asimov's famous laws of robotics. Following Asimov's first law: “a robot may not harm a human or, through inaction, allow a human to come to harm,” our ethical robot will act proactively when it anticipates (a) that a proxy-human robot is in danger of coming to harm and (b) the ethical robot can itself intervene. We have experimentally tested such a minimally ethical robot initially with e-puck robots (Winfield et al., 2014) and subsequently with NAO humanoid robots (Vanderelst and Winfield, 2018). As in the corridor experiment the ethical robot's CE models the proxy-human(s) as simple ballistic agents. In some experiments we have extended those TT models so that the ethical robot can, for instance, call out “danger!” and if the human robot then responds with “ok, understood” the ethical robot will change its model for that human from “irresponsible” to “responsible” and not intervene as it heads toward the danger zone. In this way the ethical robot is able to modify its belief about the proxy-human.

**Figure 5:**
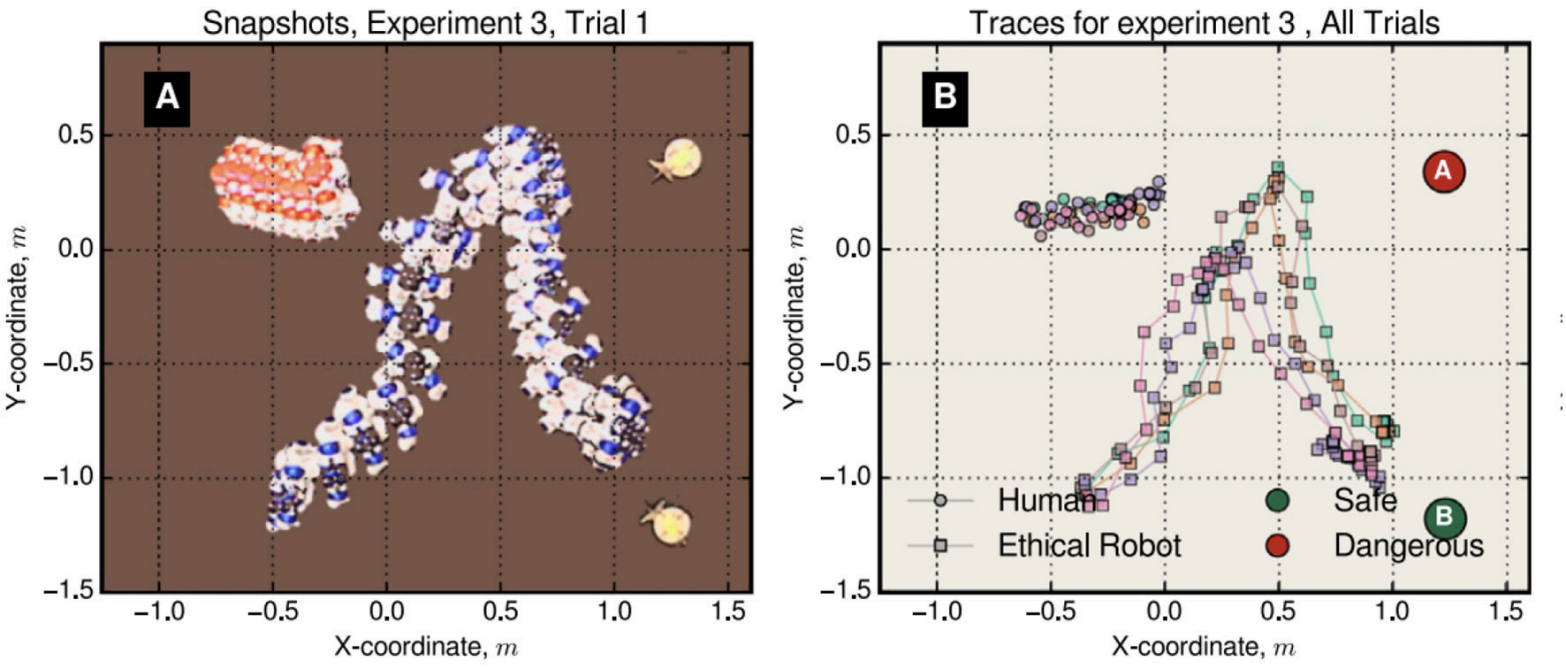
An ethical humanoid robot (blue) anticipates that proxy-human robot (red) is heading toward danger (location A at the top right). It diverts from its path toward goal position B (bottom right) to intersect red's path. Red then stops and blue resumes its path toward its goal. **(A)** Shows the trajectories of Blue and Red for trial 1. **(B)** Shows all 5 experimental trials. Figure from Vanderelst and Winfield (2018).

Figure 5 shows results from one trial with two NAO humanoid robots, one (blue) equipped with a CE and ethical logic layer, and the other (red) programmed only with short range obstacle avoidance behavior to act as a proxy-human. Figure 5 shows that the ethical robot does indeed reliably intervene, diverting from its own path, and when red halts to avoid a collision with blue, blue then continues toward its own goal.

**Figure 6:**
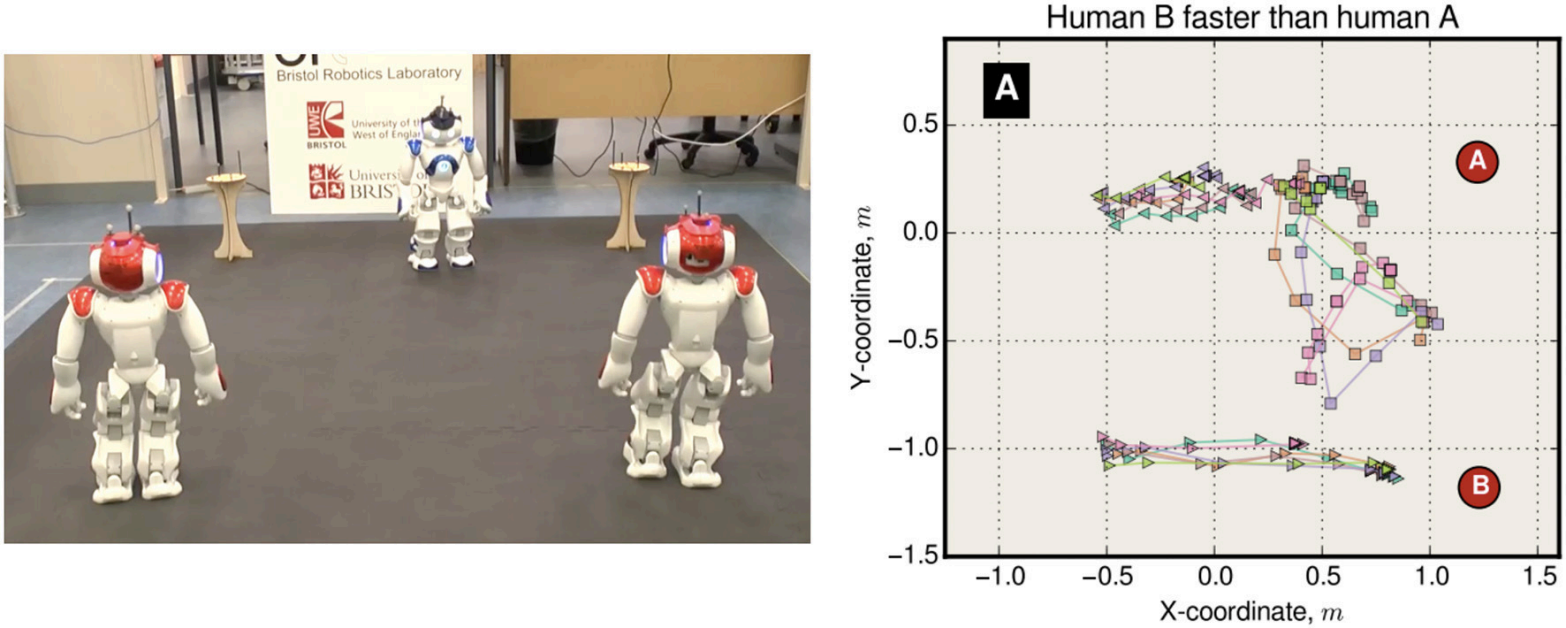
An ethical dilemma. **Left**: The ethical robot is initially positioned midway between and slightly to the front of two danger zones A and B. **Right**: The ethical robot's trajectories are shown here plotted with squares. Two proxy-human robots start from the left, both heading toward danger—trajectories plotted with triangles. Results of 5 trials are shown here.

We have tested the same ethical robot (running identical code) in a scenario with two proxy-humans both heading toward danger at the same time. These trials, first with e-puck robots (Winfield et al., 2014) and more recently with NAO robots, are believed to be the first experimental tests of a robot facing an ethical dilemma. We did not provide the ethical robot with a rule or heuristic for choosing which proxy-human to “rescue” first, so that the ethical robot faces a balanced dilemma. Figure 6 (left) shows the experimental arena with the ethical robot (blue) initially equidistant from the two (red) proxy-human robots. The trajectory plots in Figure 6 (right) interestingly show that in three of the five trials blue initially chose to move toward the red robot heading toward danger (B), but then appeared to ‘change its mind' to “rescue” the other red robot. Exactly the same “dithering” emergent behavior was observed with the e-puck robots in Winfield et al. (2014), and can be explained in part by the fact that the ethical robot's CE is running continuously, re-evaluating the consequences of its own and the other robots' behaviors and perhaps choosing a new action once per second^5^. This makes our ethical robot pathologically indecisive.

### 3.3. The imitation of goals

The imitation of goals is a very important form of social learning in humans. This importance is reflected in the early emergence of imitation in human infants; from the age of two, humans can imitate both actions and their intended goals (Gariépy et al., 2014) and this has been termed rational imitation.

Imitation has long been regarded as a compelling method for (social) learning in robots. However, robot imitation faces a number of challenges (Breazeal and Scassellati, 2002). One of the most fundamental issues is determining what to imitate (Carpenter et al., 2005). Although not trivial it is relatively straightforward to imitate actions, but inferring goals from observed actions and thus determining which parts of a demonstrated sequence of actions are relevant, i.e., rational imitation, is a difficult research problem.

The approach we explore in Vanderelst and Winfield (2017), is to equip the imitating robot with a simulation-based internal model that allows the robot to explore alternative sequences of actions required to attain the demonstrator robot's potential goals (i.e., goals that are possible explanations for the observed actions). Comparing these actions with those observed in the demonstrator robot enables the imitating robot to infer the goals underlying the observed actions.

**Figure 7:**
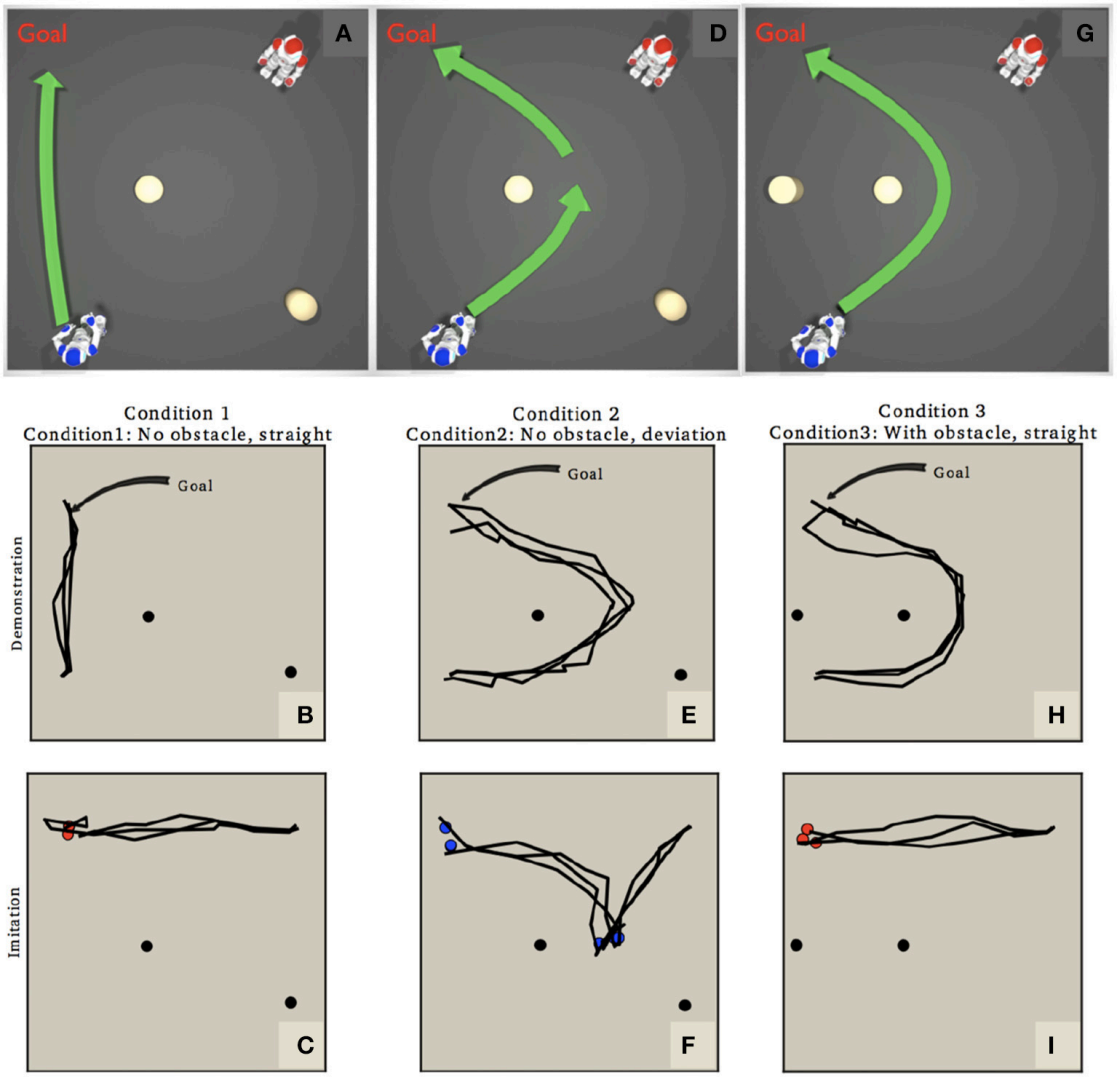
Rational imitation. **(A,D,G)** Show the setup with blue as the demonstrating robot and red the observing (then imitating) robot. In condition 1 **(A,B,C)** blue moves directly to its goal position. In condition 2 **(D–F)** blue diverts around an obstacle even though it could move directly to its goal position. And in condition 3 **(G–I)** blue's path is blocked so it cannot go directly to its goal. **(B,E,H)** Show trajectories of 3 runs of the demonstrator robot blue, and **(C,F,I)** Show trajectories of 3 runs of the imitating robot red. Note that red starts from the position it observes from. Figures from Vanderelst and Winfield (2017).

Figure 7 shows one of several experiments from Vanderelst and Winfield (2017). Here the red robot imitates the goals of the blue robot. In condition 1 blue moves directly to its goal position (Figures 7A,B). Blue infers the goal is to move to red's goal and does so directly in Figure 7C. In condition 2 blue deviates around an obstacle even though it has a direct path to its goal (Figures 7D,E). In this case red infers that the deviation must be a sub-goal of blue—since blue is able to go directly to its goal but chooses not to—so in Figure 7F red creates a trajectory via blue's sub-goal. In other words red has inferred blue's intentions to imitate its goals. In condition 3 blue's path to its goal is blocked so it has no choice but to divert (Figures 7G,H). In this case red infers that blue has no sub-goals and moves directly to the goal position (Figure 7I).

### 3.4. An embodied computational model of storytelling

Consider the idea that some of the what-if sequences tested with a robot's consequence engine are constructed fictions, i.e., “if I had turned left I would have collided with a wall.” While others—the ones actually enacted—could be historical narratives, i.e., “I turned right and reached my goal.”

Assume that we have two robots, each equipped with the same simulation-based internal model of Figure 1. Let us also assume that the robots are of a similar type, in other words they are conspecifics. Let us now extend the robots' capabilities in the following way. Instead of simply discarding (“forgetting”) an action that has been modeled, the robot may transmit that action and its predicted or actual consequences to another robot.

**Figure 8:**
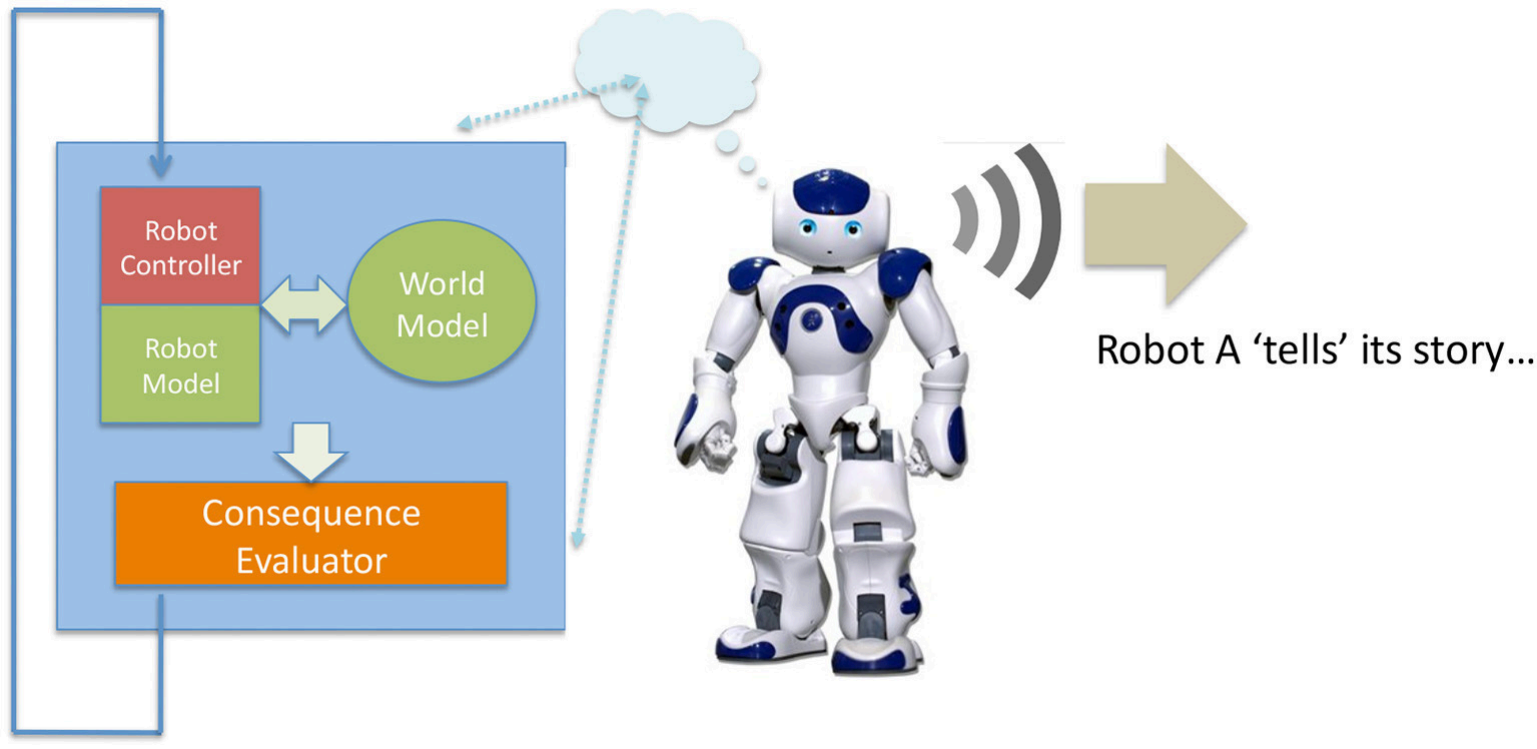
Robot A, the storyteller, “narrativizes” one of the “what-if” sequences generated by its generate-and-test machinery. First an action is tested in the robot's internal model (left), second, that action—which may or may not be executed for real—is converted into speech and spoken by the robot. From Winfield (2018).

Figure 8 illustrates robot A “imagining” a what-if sequence, then narrativizing that sequence. It literally signals that sequence using some transmission medium. Since we are building a model and it would be very convenient if it is easy for human observers to interpret the model, let us code the what-if sequence verbally and transmit it as a spoken language sequence. Technically this would be straightforward to arrange since we would use a standard speech synthesis process. Although it is a trivial narrative robot A is now able to both “imagine” and then literally tell a story. If that story is of something that has not happened it is a fictional narrative, otherwise it is a historical narrative.

**Figure 9:**
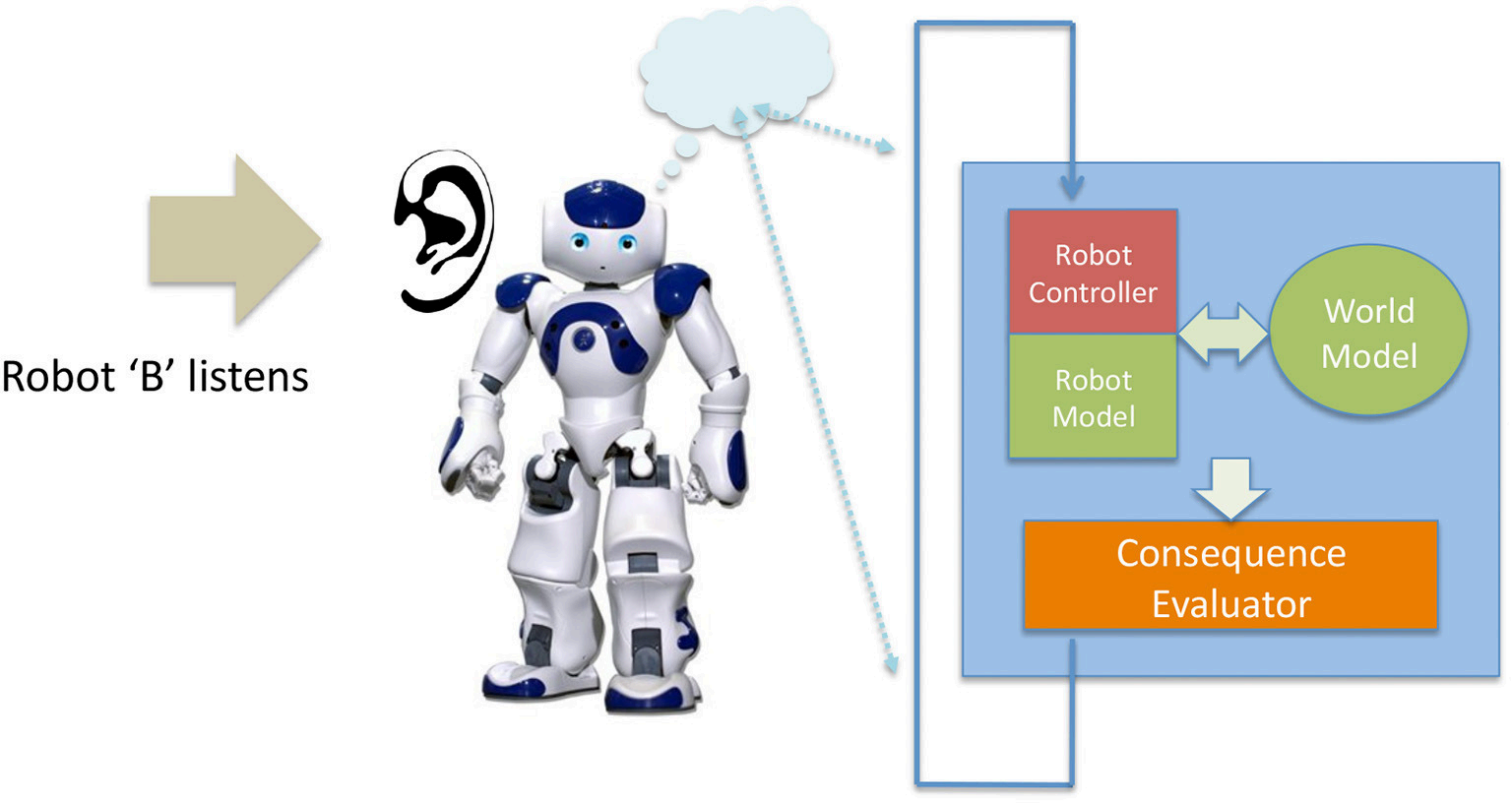
Robot B, the listener, uses the same “what-if” cognitive machinery to “imagine” robot A's story. Here the robot hears A's spoken sequence, then converts it into an action which is tested in B's internal model. From Winfield (2018).

Robot B is equipped with a microphone and speech recognition process it is thus able to “listen” to robot As story, as shown in Figure 9. Let us assume it is programmed, so that a word used by A signifies the same part of the what-if action sequence to both A and B. Providing the story has been heard correctly then robot B will interpret robot A's story as a what-if sequence. Now, because robot B has the same internal modeling machinery as A- they are conspecifics- it is capable of running the story it has just heard within its own internal model. In order that this can happen we need to modify the robots programming so that the what-if sequence it has heard and interpreted is substituted for an internally generated what-if sequence. This would be easy to do. But, once that substitution is made, robot B is able to run A's what-if sequence (its story) in *exactly* the same way it runs its own internally generated next possible actions, simulating and evaluating the consequences. Robot B is therefore able to “imagine robot A's story^6^.

In this model we have, in effect, co-opted the cognitive machinery for testing possible next actions for “imagining,” or introspectively experiencing, heard stories. By adding the machinery for signaling and signifying internally generated sequences (narratives)—the machinery of semiotics—we have constructed an embodied computational model of storytelling.

A major problem with human-robot interaction is the serious asymmetry of theory of mind (Winfield, 2010). Consider an elderly person and her care robot. It is likely that a reasonably sophisticated near-future care robot will have a built-in (TT) model of an elderly human (or even of a particular human). This places the robot at an advantage because the elderly person has no theory of mind at all for the robot, whereas the robot has a (likely limited) theory of mind for her. Actually the situation may be worse than this, since our elderly person may have a completely incorrect theory of mind for the robot, perhaps based on preconceptions or misunderstandings of how the robot should behave and why. Thus, when the robot actually behaves in a way that doesn't make sense to the elderly person, her trust in the robot will be damaged and its effectiveness diminished (Stafford et al., 2014).

The storytelling model proposed here provides us with a powerful mechanism for the robot to be able to generate *explanations* for its actual or possible actions. Especially important is that the robot's user should be able to ask (or press a button to ask) the robot to explain “why did you just do that?” Or, pre-emptively, to ask the robot questions such as “what would you do if I fell down?” Assuming that the care robot is equipped with an autobiographical memory^7^, the first of these questions would require it to re-run and narrate the most recent action sequence to be able to explain why it acted as it did, i.e., “I turned left because I didn't want to bump into you.” The second kind of pre-emptive query requires the robot to interpret the question in such a way it can first initialize its internal model to match the situation described, run that model, then narrate the actions it predicts it would take in that situation. In this case the robot acts first as the listener in Figure 9, then as the narrator in Figure 8. In this way the robot would actively assist its human user to build a theory-of-mind for the robot.

## 4. Discussion

### 4.1. Related work

One of the most influential works to date on proposing and implementing artificial theory of mind is Scassellati's 2002 paper *Theory of Mind for a Humanoid Robot* (Scassellati, 2002). Based on aspects of theory of mind present in young (4 month old) infant humans the author describes an implementation of visual attention, finding faces and the recognition and tracking of eyes, and discrimination between animate and inanimate, on the MIT Cog robot (Brooks et al., 1999). In contrast with the present work Scassellati (2002) is based on theory theories of mind (TT). Other works have also explored the important role of shared attention in social interaction and development, for instance Deák et al. (2001) and Kaplan and Hafner (2006).

Kim and Lipson (2009) describe an approach in which one robot uses an ANN to learn another's intentions based on its behavior. A very recent paper *Machine Theory of Mind* also describes a machine learning approach in which one agent observes another's behaviors and learns a predictive model of that agent (Rabinowitz et al., 2018); the simulated agents of this work learn the rules underlying the behavior of the observed agent, hence this is also a TT approach.

Several authors have proposed artificial theory of mind as a mechanism for improved human-robot interaction. Devin and Alami (2016), for instance, describe an implementation in which a robot estimates the status of the goals of a human with which it is interacting (i.e., “in progress,” “done,” “aborted” or “unknown”). Görür et al. (2017) also propose a mechanism for estimating a human's beliefs about possible actions in a shared human-robot task; they propose a stochastic approach in which a Hidden Markov Model estimates action states in the set (“not ready,” “ready,” “in progress,” “help needed,” “done,” and “aborted”).

A number of authors focus on the role of deception as an indicator of theory of mind. Terada and Ito (2010) outline an experiment to deceive a human about the intentions of a robot, noting that the experimental result indicated that unexpected change of a robot behavior gave rise to an impression of being deceived by the robot. Wagner and Arkin (2011) describe an experiment in which two robots play a game of hide and seek in which one, the hider, attempts to deceive the seeker by sending false information.

A small number of works have also proposed “like-me” or “self-as” simulation approaches, including Kennedy et al. (2009) and Gray and Breazeal (2014). Kennedy et al. (2009) promote like-me simulation as “a powerful mechanism because for any “individual” strategy the agent has, it can reason about another agent having that strategy and, further, by creating hypothetical situations …it can predict the actions it would take under hypothetical conditions;” the paper describes a like-me simulation based on the ACT-R/E (Adaptive Control of Thought-Rational/Embodied) architecture, with two robots in which one acts as a proxy human. Gray and Breazeal (2014) describe a very elegant experiment in which a robot simulates both its own possible actions and a human's likely perception of those actions in order to choose actions that manipulate the human's beliefs about what the robot is doing — and thereby deceive the human. These two works model the simulation theory of mind (ST) and are therefore of particular relevance to the present paper.

### 4.2. Discussion and conclusions

To what extent do any of the experiments outlined in this paper demonstrate (artificial) theory of mind, as variously defined in section 2.1? We can certainly be clear about which aspects of theory of mind we cannot emulate. Our robots do not “know about minds” (Roberts, 2001) (arguably they do not know about anything), but we would also suspect that while animals have minds they too do not know about them. Nor do our robots either have, or model, affective states. And we can be quite sure that none of the robots described in this paper would pass Premack and Woodruff (1978)'s famous tests which controversially demonstrated that chimpanzee have theory of mind.

Many accounts of theory of mind are couched in terms of modeling or predicting the “mental states” of others (Astington and Dack, 2008; Birch et al., 2017), but there are two problems with the use of this term. The first is that there is no clear understanding or agreement over what mental states are in animals and humans; it seems that the term is used as a proxy for several things including beliefs, desires, emotions and intentions. Secondly, robots are not generality regarded as having mental states. They certainly do not have emotions, but they arguably can have a machine analog of simple beliefs (i.e., that the path to the left is safe, whereas the path to the right is unsafe, or a belief that another agent is moving toward danger and that by inference its mental state is “unaware of danger”), simple desires (i.e., to maintain its energy level by returning to a recharging station whenever its battery charge drops below a certain level) and intentions (i.e., goals, such as “navigate safely to position x”). Although we have not used the term mental states in this paper nor do the experiments of this paper explicitly label such states they can be properly described as predicting and/or inferring the beliefs, desires and intentions of both themselves and others.

If we accept simulation of self and other as an artificial analog of mental representation, then our robots do demonstrate this attribute. The experiments of sections 3.1 and 3.2 show that a robot with a simulation-based internal model is capable of predicting the consequences of its actions for both itself and one or more robots acting as proxy humans, and choosing actions on the basis of either safety or ethical considerations. They can therefore “reason about,” i.e., model, the intentions of others, even though those models are very simple ballistic TT models and, in the case of the ethical robot experiments in section 3.2, also modeled by default as irresponsibly unaware of danger. Of course our robots have a much better model of themselves than others—but is that not also true of human theory of mind? For sure we have detailed models for those close to us—family and close friends—but our models of strangers, when walking on a sidewalk for example, can be very simple (Helbing and Molnar, 1995).

Although it is an unsophisticated example, arguably the pedestrian experiment in section 3.1 demonstrates false beliefs when each models the other as continuing in a straight line when, in fact, they each turn into the other's path (Figure 4, right). In fact we have also shown that it is surprisingly easy to turn an ethical robot into a mendacious (deceptive) robot, so that it behaves either competitively or aggressively toward a proxy human robot (Vanderelst and Winfield, 2016).

We have also demonstrated, in section 3.3, that a robot with a simulation-based internal model can infer the goals of another robot, therefore learning the other robot's intentions. Imitation is a powerful form of social learning and we argue that the inferential learning of section 3.3 demonstrates another key component of theory of mind.

The model of storytelling proposed in section 3.4 gets, we contend, to the heart of theory of mind. Theory of mind works best between conspecifics: in general you can much better understand your partner's beliefs and intentions than your cat's. The two robots in our thought experiment of section 3.4 would in principle be able to learn each other's beliefs and intentions in a very natural (to humans) way, through *explanation*. This is, after all, one of the key mechanisms by which infant humans learn theory of mind; one only has to think of a child asking “Mummy why are you angry with me?” (Ruffman et al., 2002).

The robots of this paper all have the cognitive machinery to predict their own behavior. But we must not assume that because a robot can predict its own behavior it can predict the behavior of any other agent. Of course when those others are conspecifics then predicting the behavior of others ‘like me' becomes a (conceptually) straightforward matter of co-opting your own internal model to model others'. In all of the experiments of this paper we make use of homogeneous robots, which clearly share the same architecture (although in some cases those robots are programmed to behave differently, as proxy humans for instance). In a heterogeneous multi-robot system a robot might need to model the beliefs or intentions of a robot quite unlike itself, and the same is clearly true for a robot that might need to model the mental states of a human. But as Gray and Breazeal (2014) assert “Humans and robots, while vastly different, share a common problem of being embodied agents with sensory motor loops based on affecting and observing the physical world around them. By modeling a humans connection between mental states and the world as similar to its own, and reusing those mechanisms to help evaluate mental state consequences” a robot can at the basic level of actions and their consequences—model a human. The same is clearly also true for a robot modeling another robot of a different kind, providing that both observably sense and act in the physical world.

In the context of human-robot interaction we must consider the important problem of how a human builds a theory of mind for a robot; this could be especially important if that robot has the function of companion or elder-care (assisted living) robot. In the thought experiment of section 3.4 we outline how a robot's self-model can allow the robot to explain itself and hence assist a human to acquire an understanding of how and why the robot behaves in different circumstances.

The main contributions of this paper have been to (1) advance the hypothesis that simulation-based internal models represent a computational model of the simulation theory of mind (ST) and (2) to show that such a computational model provides us with a powerful and realizable basis for artificial theory of mind. We have shown that experiments with simulation-based internal models demonstrate the ability to predictively model the actions of both self and other agents. As summarized in Table 1 the experiments of section 3 have demonstrated both ST+TT (hybrid) and ST+ST modes for self + other, as defined in section 2.3.

**Table 1:** Table summarizing the contribution of each of the experiments of section 3 together with their respective theory modes (as defined in section 2.3).

**Experiment**	**Figures**	**Theory mode (section 2.3)**	**Notes**
Corridor experiment	2	ST+TT	One robot with ST model of self and ballistic TT model of five other robots, demonstrates predictive modeling of self and reasoning about the intentions of others, and attention radius.
Pedestrian experiment	4	ST+TT	Two robots, each with ST model of self and ballistic TT model of other, demonstrates false beliefs.
Ethical robot experiments	5 & 6	ST+TT	One robot with ST model of self and ballistic TT model of one or two other robots. Demonstrates predictive modeling of self and reasoning about the intentions of others. Ballistic TT model extended so that the ethical robot can test and modify its belief about the proxy-human.
Imitation of goals	7	ST+ST	Imitating robot uses ST to model both itself and the demonstrator robot, in order to infer the demonstrator's goals.
Story-telling robots	8 & 9	ST+ST	Storytelling robot narrates *what-if* episode from its ST model; listener robot uses its ST model to introspectively ‘imagine' that story. Potential to *explain* the past and possible future actions of self.

In summary, we contend that the experimental work outlined in this paper does demonstrate a number of components of theory of mind and can reasonably be described as “experiments in artificial theory of mind.” The main hypothesis of this paper, that simulation-based internal modeling can form the basis for artificial theory of mind has, we argue, been demonstrated. Whilst far from a complete solution, we propose simulation-based internal modeling as a powerful and interesting starting point in the development of artificial theory of mind.

## Author contributions

The author confirms that he is the sole creator of the text of this paper, and has approved it for publication.

### Conflict of interest statement

The author declares that the research was conducted in the absence of any commercial or financial relationships that could be construed as a potential conflict of interest.

## Footnotes

^1^Second order interactions are between robot and environment and third order are robot-robot interactions.

^2^While noting that having a theory of mind does not make an agent ethical

^3^There are also a number of hybrid theories which combine elements of TT and ST.

^4^Noting that even our computational model of ST is not completely theory free, since the world model models the physics of collisions, etc.

^5^In practice the ethical NAO robot would also favor the slower of the two proxy-humans, as a side-effect of its action-selection logic.

^6^Where is the meaning? It could be argued that when the listener replays the story in its internal model (functional imagination) that *is* meaning.

^7^It would be relatively easy for a robot to build a memory of everything that has happened to it, but of much greater interest here is to integrate the autobiographical memory into the internal model, perhaps leading to what Conway (2005) describes as a self-memory system (SMS).
